# Analgesic Drugs and COVID-19

**DOI:** 10.3390/jcm10030545

**Published:** 2021-02-02

**Authors:** Giustino Varrassi

**Affiliations:** Paolo Procacci Foundation, Via Tacito 7, 00193 Roma, Italy; giuvarr@gmail.com

COVID-19 pandemic represents a big challenge for the health care systems [1,2]. Mainly, it affects some medical specialties like anesthesiology [3,4] and is causing occupational burnout in many health-care professionals [5]. COVID-19 may be responsible for causing neurological damages [6] and has generated discussion about vaccines [7]. As with many critical situations, humanity has learned a lot from this experience [8], and it is continually looking for new solutions to cope with this global health-care problem.

COVID-19 is having a tremendous impact on the treatment of patients, especially those with chronic pain [9]. As a potential solution, research into remote (eHealth) treatment for the management of such patients has started [10]. The prevalence of chronic pain in primary care is definitely relevant [11]; hence, it deserves a great deal of attention both from the clinical and organizational point of view. One of the most intriguing aspects of pain treatment for the health care system under such difficult conditions is the need to prescribe analgesics. This is difficult from the organizational point of view, and it has generated much criticism and discussion. As an example, at the beginning of March 2020, *Le Figaro*, a French newspaper, reported and emphasized the assertion of some French politicians on the need to be careful with the use of NSAIDs as antifebriles because of a potential interaction with the coronavirus that might increase the risk of infection. Instead, they suggested to prefer paracetamol [12]. Very rapidly, this information spread around the world, with relevant and significant consequences, showing that the influence of the media is one of the most intriguing aspects of this pandemic [8]. Many scientists produced important publications on the abovementioned assertion by resorting to incredible and convoluted scientific justifications [13]. One of those articles was immediately criticized for its inconsistency [14,15,16,17]. The authors were obliged to completely revise their opinion in a subsequent article, but the effort was futile [18]. This topic was recently reviewed, focusing on knowns and unknowns [19], but it still deserves a lot of attention, especially because it is very important from the clinical point of view. Of course, it is important in relation to the previously described example of NSAIDs use, but it is even more important for other aspects of prescribed and OTC analgesics. This is the main reason that the “special issue” of *Journal of Clinical Medicine* was launched. We hope that open and transparent scientific information will help to clarify all aspects of this topic. It would definitely increase the quality of care of pain patients without incurring the risk of confusing and unsupported information.
